# Alterations in Coagulation and Endothelial Function in Nephrotic Syndrome: A Multicenter, Cross-Sectional Analysis

**DOI:** 10.34067/KID.0000000865

**Published:** 2025-06-06

**Authors:** Sarah Kelddal, Erik L. Grove, Camilla L. Duus, Louis B. Nygaard, Tilde Kristensen, Frank H. Mose, Jon W. Gregersen, Anne-Mette Hvas, Henrik Birn

**Affiliations:** 1 Department of Renal Medicine, Aarhus University Hospital, Aarhus, Denmark; 2 Department of Biomedicine, Faculty of Health, Aarhus University, Aarhus, Denmark; 3 Department of Cardiology, Aarhus University Hospital, Aarhus, Denmark; 4 Department of Clinical Medicine, Faculty of Health, Aarhus University, Aarhus, Denmark; 5 University Clinic in Nephrology and Hypertension, Gødstrup Hospital, Herning, Denmark; 6 Department of Nephrology, Aalborg University Hospital, Aalborg, Denmark; 7 Medical Diagnostics Center, Viborg Regional Hospital, Viborg, Denmark; 8 Faculty of Health, Aarhus University, Aarhus, Denmark

**Keywords:** GN, nephrotic syndrome, thrombosis

## Abstract

**Key Points:**

- Nephrotic syndrome is associated with excessive thrombin generation and impaired fibrinolysis, increasing the risk of thrombosis.
- Endothelial dysfunction in nephrotic syndrome is markedly by elevated thrombomodulin, syndecan-1, and vWf levels.
- Findings suggest anticoagulant therapy targeting secondary hemostasis over platelet agents in nephrotic syndrome management.

**Background:**

Nephrotic syndrome is associated with an increased risk of venous thromboembolism, but the underlying mechanism remains incompletely understood. The efficacy of prophylactic anticoagulant strategies is also poorly documented. To optimize preventive therapy, this study aimed to characterize the coagulation abnormalities in patients with nephrotic syndrome.

**Methods:**

This Danish multicenter, cross-sectional study included 47 adult patients with nephrotic syndrome, defined by plasma-albumin <30 g/L and urine albumin-creatinine ratio >2200 mg/g. Exclusion criteria included eGFR <30 ml/min per 1.73 m^2^, ongoing anticoagulant treatment, thrombophilia, prior thrombosis, malignancy, or pregnancy. Markers of endothelial cell function, platelet function, thrombin generation, and fibrinolysis were compared with those of healthy individuals (*n* ranging from 10 to 174 depending on assay).

**Results:**

Patients with nephrotic syndrome (*N*=47) had significantly increased thrombin generation markers compared with healthy individuals, as indicated by elevated prothrombin fragment 1+2 (509 pmol/L, 95% confidence interval [CI], 426 to 592 versus 183 pmol/L; 95% CI, 171 to 196; *P* < 0.001) and thrombin-antithrombin complex (3.5 *µ*g/L, 95% CI, 3.2 to 3.8 versus 2.5 *µ*g/L; 95% CI, 2.3 to 2.7; *P* < 0.001). Fibrinolysis was impaired, as demonstrated by prolonged 50% clot lysis time (1281 seconds, 95% CI, 1101 to 1462 versus 964 seconds; 95% CI, 843 to 1085; *P* = 0.004). Endothelial cell markers, including thrombomodulin, syndecan-1, and vWf, were significantly elevated. By contrast, platelet function, natural anticoagulants, and other coagulation markers did not differ.

**Conclusions:**

Our results indicate that the prothrombotic state in nephrotic syndrome is driven by excessive thrombin generation and impaired fibrinolysis rather than increased platelet aggregation. In line with current guideline recommendations, this supports the rationale for antithrombotic strategies targeting secondary hemostasis rather than platelet function in patients with nephrotic syndrome.

**Clinical Trial registry name and registration number:**

European Union Drug Regulating Authorities Clinical Trials Database (Identifier: 2019-001212-29), ClinicalTrials.gov (Identifier: NCT04850378).

## Visual Abstract

**Figure:**
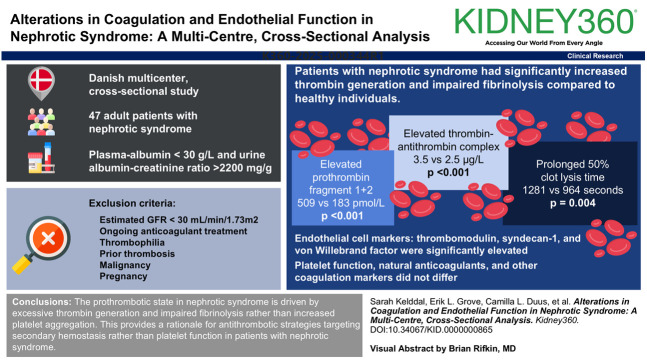

## Introduction

Nephrotic syndrome (NS) is associated with potentially life-threatening venous thromboembolic (VTE) complications.^1,2^ Despite increased focus on anticoagulant prophylaxis in recent decades, patients with NS remain at a considerable risk for VTE.^1,3,4^

The reported incidence of VTE varies considerably, with estimates ranging from approximately 1%–44%, and an average incidence of around 25% across various studies.^1,3,4^ This variation is likely due to multiple factors, including the period in which the studies were conducted as older studies generally report a higher risk,^3^ while more recent studies indicate a lower risk.^5^ Risk factors for VTE in patients with NS include the underlying glomerular disease, with membranous nephropathy presenting the highest risk,^5^ hypoalbuminemia, particularly when plasma albumin levels fall below 20 g/L,^6^ the initial 3–6 months after the diagnosis of NS,^7^ and patient age with children demonstrating the lowest incidence.^1^

Several studies, most of which are small and conducted in earlier years, have explored changes in the coagulation system in NS patients to explain their elevated risk of VTE and to optimize prophylactic treatment.^8–14^ These studies report various abnormalities in NS, including endothelial cell activation and injury, increased platelet aggregation, antithrombin deficiency due to urinary losses, high thrombin generation, and impaired fibrinolytic activity. Importantly, no previous study has simultaneously assessed endothelial cell injury, platelet abnormalities, natural anticoagulants, thrombin generation, and fibrinolytic activities within the same well-characterized cohort of patients with NS. Given the complex and multifactorial nature of the hypercoagulable state in NS, such integrated evaluation may provide a better understanding of the abnormalities in different pathways and guide the exploration of future preventive strategies. Furthermore, the effect of prophylactic anticoagulant therapy in NS remains poorly understood. Current guidelines recommend low molecular weight heparin (LMWH) or warfarin for patients at high VTE risk and low risk of bleeding. For patients at high risk of bleeding, aspirin is suggested as an alternative.^15^ However, evidence supporting the use of LMWH and warfarin remains limited to small retrospective studies, and the associated bleeding risk continues to raise concerns.^16–20^ Aspirin has been proposed as an alternative in patients with high bleeding risk, although reduced efficacy has been reported in NS.^21^ Notably, a substantial proportion of patients are already receiving aspirin at the time of VTE.^6^ These uncertainties highlight the need for better mechanistic understanding of the prothrombotic state in NS to facilitate investigation of novel prophylactic strategies.

To improve our understanding and provide a stronger rationale for prophylactic anticoagulant treatment, we aimed to provide a comprehensive analysis of the coagulation profile and endothelial markers in patients with NS compared with healthy individuals.

## Methods

### Design

This was a Danish multicenter cross-sectional investigation. The study was approved by the Danish Medicines Agency (Reference No. 2020061178) and the Danish Research Ethics Committees (Reference No. 1-10-72-158-20). It was registered with ClinicalTrials.gov (Identifier: NCT04850378, registered on March 25, 2021) and European Union Drug Regulating Authorities Clinical Trials Database (Identifier: 2019-001212-29, registered on August 26, 2021). All study procedures followed the principles of the Declaration of Helsinki, and written informed consent was obtained from each participant before any trial-related procedures. The study was monitored by the Good Clinical Practice unit at Aarhus University and Aalborg University, Denmark.

### Patient Population and Study Protocol

Participants with newly diagnosed NS or a relapse of NS were enrolled at four nephrologic departments across Denmark from April 2021 to May 2024. Eligibility criteria included plasma albumin levels below 30 g/L, albuminuria with urine albumin-creatinine ratio (uACR) >2200 mg/g, and 18 years or older. Albumin constitutes about 65% of total urinary protein excretion in glomerular disease.^15^ Thus, a threshold of 2200 mg/g corresponds to approximately 3.5 g/d of total protein, making uACR a reasonable surrogate when 24-hour collection and/or protein-to-creatinine ratio are not available. Indeed, this definition has been applied in other studies.^22^

Exclusion criteria were an eGFR < 30 ml/min per 1.73 m^2^, uncontrolled diabetes defined by hemoglobin A1c ≥65 mmol/mol, anticoagulant or antiplatelet treatment for comorbidities, use of nonsteroidal anti-inflammatory drugs, known coagulation disorder, prior VTE, clinical infection, malignancy, or pregnancy. Following consent, blood, urine, and relevant clinical information were collected.

A systematic screening log was not maintained, and we are therefore unable to specify the number of patients excluded based on individual criteria.

### Reference Groups for Healthy Individuals

Different groups of healthy individuals were used for comparative analysis depending on the specific assay. Laboratory-established reference values from previous studies were applied for thrombin generation marker, including endogenous thrombin potential (ETP), prothrombin fragment 1+2 (F1+F2), and thrombin-antithrombin (TAT) complex.^23^ For platelet function, previously published reference values were used for thrombin receptor-activating peptide (TRAP) aggregation, ADP aggregation and ASPI aggregation,^24^ and serum thromboxane B_2_ (TXB_2_; Supplemental Figure 1).^25^ The reference values used for comparison were derived from study using the same assay platforms as those applied in this study, ensuring methodologic consistency and comparability of results.

For endothelial markers, reference values for thrombomodulin, Syndecan-1, and sE-selectin were obtained from kit-based references reported by the manufacturers without age or sex specifications.^26–28^ For 50% clot lysis time, healthy blood donors were selected as a reference group. Frequency matching was applied based on predefined age strata (*e.g*., 40–44 years) and overall sex distribution to reflect the demographic characteristics of the NS cohort. No individual one-to-one matching was performed. In addition, vWf levels were compared with ten healthy individuals included in this study. Standard hospital reference intervals were applied for routine blood tests.

As clinical data such as age and sex were only available for selected reference groups, regression-based comparisons were not performed between patients and controls, and adjusted analyses were therefore limited to NS cohort.

### Demographic and Clinical Data Collection

Information on age, sex, body mass index (BMI), comorbidities, previous VTE, and bleeding episodes were collected from electronic medical records and patient interviews.

### Coagulation Profile and Thrombin Generation Markers

The primary outcomes were thrombin generation markers and fibrinolytic activity, while secondary outcomes included endothelial and platelet function.

Thrombin generation was assessed using both *in vivo* and *ex vivo* markers. *In vivo* thrombin generation was evaluated by measuring F1+F2 and TAT, which reflect ongoing thrombin activation in the circulation. *Ex vivo* thrombin generation was assessed using ETP, which measures the plasma's ability to generate thrombin under standardized conditions.^29^

### Laboratory Analyses

#### Thrombin Generation Markers

Blood samples for thrombin generation markers ETP, F1+F2, and TAT were collected in tubes containing 3.2% sodium citrate. Following collection, samples were centrifuged at 3000×*g* for 25 minutes at 20°C to obtain platelet-poor plasma, which was subsequently aliquoted and stored at −80°C until batch analysis. F1+F2 levels were determined using an ELISA technique with the Enzygnost F1+F2 Mono assay (Siemens Healthcare GmbH), while TAT levels were measured with the Enzygnost TAT essay (Siemens Healthcare Diagnostics, Marburg, Germany).^23^ The results with a coefficient of variation exceeding 10% were reanalyzed, and TAT concentrations below the detection limit of 2 *µ*g/L were assigned a value of 1 *µ*g/L. For ETP measurements, an additional centrifugation step at 2500×*g* for 15 minutes at 20°C was performed before storage at −80°C. ETP was determined in platelet-poor plasma using calibrated automated thrombograms (Thrombinscope, Maastrict, The Netherlands),^23^ with the area under the curve (nM×minutes) as the end point.

#### Fibrinolytic Markers

Blood samples for fibrinolytic markers, including 50% clot lysis time, were processed in the same manner as thrombin generation markers, with plasma stored at −80°C until analysis. Clot lysis time was assessed using a locally developed in-house assay following an established protocol.^30^

#### Endothelial Cell Markers

Blood samples for endothelial cell markers, including thrombomodulin, syndecan-1, and sE-selectin, were collected in citrate tubes (3.2% sodium citrated) and centrifuged at 3000×*g* for 25 minutes at 20°C. Plasma was aliquoted and stored at −80°C until analysis. Endothelial cell markers were quantified using ELISA. Trombomodulin was quantified with Human sCD141 ELISA kit (Diaclone, France), syndecan-1 with Human CD138 ELISA kit (Diaclone, France), and sE-Selectin with the (Quantikine ELISA, R&D Systems, US and Canada).

#### Platelet Function Markers

Platelet aggregation was assessed in whole blood samples collected in hirudin tubes and allowed to rest for 30 minutes before analysis. Measurements were performed using impedance aggregometry on a Multiplate 5.0 Analyzer (Roche Diagnostics GmbH, Switzerland). Platelet function was evaluated using three agonists: ADP (ADPtest, 6.5 *μ*M), AA (ASPItest, 0.5 *μ*M), and TRAP (TRAPtest, 32 *μ*M). The results were reported as area under the curve (AU×minutes).^24^ Serum TXB_2_ was quantified using an enzyme immunoassay following the manufacturer's instructions (Cayman Chemicals, Ann Arbor, MI). Before analysis, whole blood was incubated at 37°C for 30 minutes to allow colt formation, and serum was subsequently separated by centrifugation.

#### Standard Laboratory Tests

Plasma albumin and creatinine concentrations were measured using the Atellica CH analyzer (Siemens Healthcare GMBh), while complete blood counts were obtained on an XN9000 analyzer (Sysmex, Kobe, Japan). Natural anticoagulants were assessed by measuring antithrombin on the CS 5100i (Sysmex) and protein C and free protein S on the CS 2100i (Sysmex). Coagulation tests, including thrombin time, international normalized ratio, and activated partial thromboplastin time, were performed on the CS 5100i. In addition, D-dimer and fibrinogen levels were measured using the CS 5100i.

### Statistical Analyses

Baseline demographics are presented in a contingency table. Continuous variables were assessed for normality using QQ-plots and are reported as means±standard derivations or median with interquartile range (IQR; lower and upper quartiles).

Comparisons between patients with NS and the reference groups were performed using an unpaired *t* test for normally distributed data, reporting mean differences and 95% confidence interval (CI), and a Mann–Whitney *U* test for non-normally distributed data reporting median values and IQRs. When data on the individual results were available, the results are displayed in dot plots; otherwise, the results are presented as mean values with 95% CIs or medians with IQRs. Analyses were based on available data without imputation for missing values. Patients with missing data for a specific variable were excluded from that particular analysis. A two-sided *P* value was used for hypothesis testing, with a significance level of <0.05 considered indicative of statistical significance.

To correct for multiple comparison, false discovery rate (FDR) adjustment using the Benjamini-Hochberg procedure was applied. Group-wise correction was performed within predefined categories (thrombin generation, platelet function, and endothelial markers), and a global correction was applied across all biomarkers including 50% clot lysis time.

In addition, multivariable linear regression analyses were performed to adjust for potential confounders, including plasma albumin, uACR, eGFR, BMI, C-reactive protein (CRP), age, and sex.

All statistical analyses were performed using STATA software version 17.0 (StataCorp, College Station, TX), and all figures were created in GraphPad Prism version 10.3.1 (GraphPad Software, San Diego, CA).

## Results

We included 47 patients with NS, and their demographics and baseline markers are presented in Table 1. The mean age was 47 years, and the cohort comprised of slightly more men than women (55%). Apart from hypertension, no other comorbidities associated with albuminuria or hypoalbuminemia were identified. The patients exhibited severe NS, with a mean plasma albumin level of 22±5 g/L and a mean uACR of 5161±2159 mg/g. The mean eGFR was 78±25 ml/min per 1.73 m^2^ and the most common glomerular diseases were membranous nephropathy and minimal change disease constituting about two thirds of the total cohort.

**Table 1:** Demographics and baseline characteristics in patients with nephrotic syndrome

Characteristics	Patients with NS (*N*=47)
Male, *n* (%)	26 (55)
Age (yr), mean±SD	47±19
BMI (kg/m^2^), mean±SD	28.3±5.7
**Comorbidities, *n* (%)**	
Hypertension	21 (45)
Heart failure	0 (0)
Liver disease	0 (0)
**Renal diagnosis *n* (%)**	
Membranous nephropathy	15 (32)
Minimal change disease	17 (36)
FSGS	4 (9)
Proliferative GN^a^	5 (11)
Amyloidosis/myeloma	4 (9)
Other diagnosis	2 (4)
**Medication, *n* (%)**	
Glucocorticoids	11 (23)
Statins	36 (77)
ACE inhibitors/ARBs	33 (70)
Diuretics	19 (40)
**Kidney function**	
eGFR, ml/min per 1.73 m^2^, *n* (%)	
*eGFR >90*	15 (32)
*eGFR 60–90*	20 (43)
*eGFR 30–59*	12 (26)
*Plasma-albumin, g/L [reference: 36–45]*	22±5
*uACR, mg/g*	5161±2159
**Inflammatory and metabolic markers**	
Median CRP, mg/L (IQR) [reference: <8]	4.0 (3.0–4.3)
Median D-dimer, mg/L (IQR) [reference: <0.60]	0.93 (0.56–1.30)
Fibrinogen, *µ*mol/l [reference: 5.5–12.0]	16.7±4.4
Median glycated hemoglobin, mmol/mol (IQR) [reference: <48]	36 (33–38)
**Blood counts**	
Hemoglobin, mmol/l [reference: 7.3–10.5]	8.8±1.1
White blood cells, ×10^9^/l [reference: 3.5–10.0]	8.3±2.5
**Platelets**	
Median platelet count, ×10^9^/l (IQR) [reference: 145–400]	296±92
TXB_2_, ng/L (49–522)	297±191
**Natural anticoagulant**	
Antithrombin, 10^3^ IU/L [reference: 0.80–1.20]	0.94±0.20
Protein C, 10^3^ IU/L [reference: 0.74–1.50]	1.53±0.30
Protein S free, 10^3^ IU/L [reference: 0.69–1.37]	1.13±0.22
**Markers of hemostasis**	
Thrombin time, s [reference: <21]	17±1.3
Median activated partial thromboplastin time, s (IQR) [reference: 20–29]	25 (23–27)
Factor 8, 10^3^ IU/L [reference: 0.66–1.55]	2.23±0.58
Factor 10, 10^3^ IU/L [reference: 0.74–1.52]	0.97±0.19

Demographic, clinical, and laboratory baseline characteristics of patients with NS. Categorical variables are presented as numbers (*n*) and percentages (%), while continuous variables are displayed as a means with standard derivation or median and interquartile range. Reference intervals are reported in the square brackets. ACE, angiotensin-converting enzyme; ARB, angiotensin II receptor blocker; BMI, body mass index; CRP, C-reactive protein; IQR, interquartile range; IU, international units; NS, nephrotic syndrome; TXB_2_, thromboxane B_2_; uACR, urine albumin creatinine ratio.

a Proliferative GN includes membranoproliferative, mesangioproliferative, and IgA nephropathy.

### Thrombin Generation Markers

**Figure 1:**
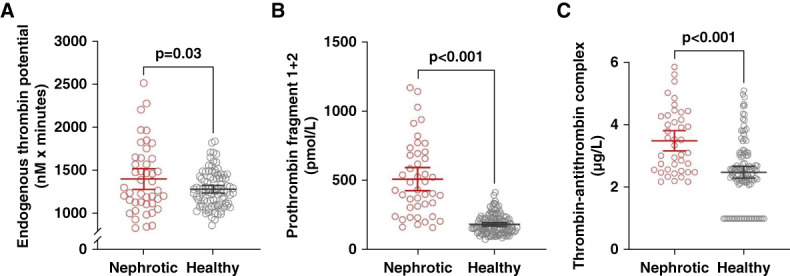
**Thrombin generation markers in patients with nephrotic syndrome compared with healthy individuals.** Thrombin generation markers in patients with NS (NS, *N*=47, red circles) and healthy individuals (gray circles). (A) ETP: healthy individuals *N*=90, (B) prothrombin F1+F2: healthy individuals *N*=127, and (C) TAT complex: healthy individuals *N*=127. Groups were compared using the unpaired *t* test. ETP, endogenous thrombin potential; F1+F2, fragment 1+2; NS, nephrotic syndrome; TAT, thrombin-antithrombin.

Both the *in vivo* thrombin generation markers, F1+F2 and TAT, as well as the *ex vivo* thrombin generation marker, ETP, were significantly elevated in patients with NS compared with healthy individuals (Figure 1 and Supplemental Table 1).

### Fibrinolytic Markers

**Figure 2:**
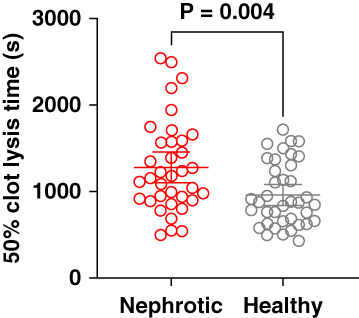
**Fifty percent clot lysis time in patients with NS compared with age-matched and sex-matched healthy individuals.** NS, *N* = 47 red circles; healthy blood donors, *N*=47, gray circles. Groups were compared using the unpaired *t* test.

Median plasma plasminogen activator inhibitor-1 levels were comparable between patients with NS and healthy individuals (8.4 ng/ml, IQR 4.6–10.4 versus 6.6 ng/ml, IQR 4.0–9.1; *P* = 0.17). By contrast, the 50% clot lysis time was significantly prolonged in patients with NS compared with age-matched and gender-matched healthy individuals (Figure 2 and Supplemental Table 1).

### Endothelial Cell Markers

**Figure 3:**
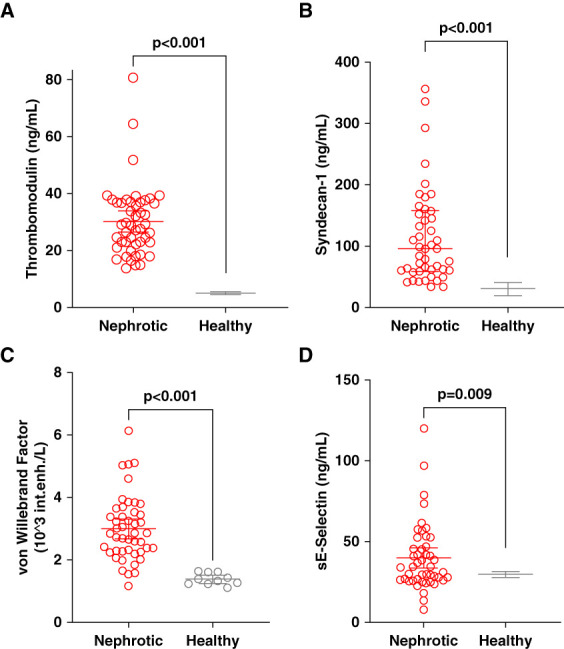
**Plasma endothelial cell markers in patients with nephrotic syndrome.** Plasma levels of endothelial cell markers in patients with NS (NS, *N*=47, red circles) compared with healthy individuals (gray, A) thrombomodulin: healthy individuals *N*=40, (B) syndecan-1: healthy individuals *N*=40, (C) sE-selectin: healthy individuals *N*=35, and (D) vWf antigen, *N*=10. Statistical comparisons were performed using the unpaired *t* test (A, C, and D) or the Mann–Whitney *U* test (B). sE, soluble E-selectin.

Compared with healthy controls, patients with NS revealed significantly elevated endothelial cell markers in plasma, including the anticoagulant receptor thrombomodulin, the glycocalyx marker syndecan-1, and the adhesion molecule sE-selectin, compared with kit-based references. In addition, vWf was markedly higher in patients with NS than in healthy individuals (Figure 3 and Supplemental Table 1).

### Platelet Aggregation

**Figure 4:**
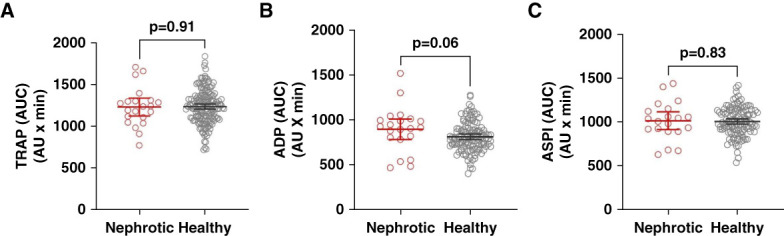
**Platelet aggregation response in patients with nephrotic syndrome compared with healthy individuals.** Platelet aggregation was assessed using impedance aggregometry and is reported as AUC values. Results in patients with nephrotic syndrome (NS, *n*=21, red circles) and healthy individuals (gray circles) are shown for: (A) TRAP: healthy individuals *N*=174, (B) ADP: healthy individuals *N*=121, and (C) ASPI: healthy individuals *N*=121. Platelet aggregation testing in NS was only available at the main center, resulting in a reduced sample size. Groups were compared using the unpaired *t* test. AUC, area under the curve; TRAP, thrombin receptor-activating peptide.

Patients with NS had normal platelet counts, and plasma levels of the *in vivo* platelet activation marker TXB_2_ were within the normal range (Table 1). Similarly, no significant differences were observed between patients with NS and healthy individuals in *ex vivo* platelet aggregation measurements induced by TRAP, ADP, and ASPI (Figure 4 and Supplemental Table 1).

### Adjusted Analysis and Correction for Multiple Comparisons

To account for potential interaction with additional determinants of coagulation dysfunction, we performed multivariable linear regression analysis for all biomarkers, adjusting for plasma albumin, uACR, eGFR, BMI, CRP, age, and sex (Supplemental Tables 2 and 3). Among patients with NS, age was significantly associated with F1+F2 (*β*=7.2 ng/L per years, 95% CI, 3.3 to 11.1; *P* = 0.001), while no other parameters were significantly correlated with age. Furthermore, lower eGFR was independently associated with increased F1+F2 and thrombomodulin. Lower plasma albumin was significantly associated with elevated syndecan-1 levels and showed trends toward increased F1+F2 and prolonged clot lysis time. Sex, uACR, BMI, and CRP were not significantly associated with any of the coagulation or endothelial markers.

Given the observed association between age and F1+F2 within the NS cohort, we performed a multivariable regression analysis comparing patients with NS to healthy individuals, adjusting for age and sex. The group difference in F1+F2 remained statistically significant (*β*=966 ng/L, 95% CI, 407 to 1516; *P* = 0.001), while neither age nor sex showed an independent association with F1+F2 levels. This supports that the elevated F1+F2 observed in NS is not attributable to age or sex differences between groups.

To further support the robustness of our results, we applied FDR correction for multiple comparisons, both within biologically related biomarker groups and across all tests. FDR-adjusted *P* values confirmed the significance of key findings and did not alter the overall interpretation (Supplemental Table 4).

## Discussion

Patients with NS exhibit a procoagulant pattern characterized by increased levels of both *in vivo* and *ex vivo* thrombin generation markers, prolonged fibrinolysis, and significantly elevated endothelial cell markers, indicating endothelial cell activation and injury. All these factors may contribute to the increased risk of VTE in patients with NS.

The procoagulant state evident from elevated levels of both *in vivo* thrombin generation markers (F1+F2 and TAT) and the *ex vivo* marker ETP is consistent with previous studies in patients with NS.^8,13,14^ Studies in cancer showed that elevated F1+F2 and TAT are consistently linked to increased risk of VTE, suggesting that *in vivo* thrombin activation plays a key role in thrombosis development.^31,32^ Thus, F1+F2 is a promising predictor of VTE, also highlighted in a recent systematic review.^32^ Although *ex vivo* thrombin generation has shown inconsistent results in cancer, the Vienna Cancer and Thrombosis Study demonstrated that higher peak thrombin was associated with an increased risk of subsequent VTE.^33^ Whether a similar mechanism applies to NS remains unclear, but our findings suggest that the concurrent elevation of both *in vivo* and *ex vivo* markers may reflect a sustained hypercoagulable state that could contribute to VTE risk in patients with NS.

In addition to increased thrombin generation, we observed impaired fibrinolytic activity, as evidenced by prolonged fibrinolysis time, which aligns with previous findings in patients with NS.^34,35^ Given that prolonged clot lysis time has been associated with an increased risk of VTE in other populations,^36,37^ the combination of increased thrombin generation and impaired fibrinolysis may contribute to a sustained prothrombotic state in patients with NS.

The underlying mechanism behind the increased thrombin generation and impaired fibrinolysis observed are likely multifactorial. Disruption of the glomerular filtration barrier leads to urinary loss of antithrombin and other coagulation proteins, tipping the balance toward coagulation activation.^38^ At the same time, hypoalbuminemia reduces plasma oncotic pressure, which stimulates hepatic synthesis of procoagulant factors, further enhancing thrombin generation.^39^ Our findings of elevated F1+F2, TAT, and ETP are consistent with such pathophysiology. uACR was not significantly associated with coagulation or endothelial markers. Although it reflects glomerular albumin loss, its relationship with systemic coagulation is likely influenced by hepatic albumin synthesis and tubular reabsorption. By contrast, plasma albumin showed stronger associations, suggesting it may better reflect the hemostatic impact of NS.

Endothelial dysfunction may arise from inflammation, dyslipidemia, hypoalbuminemia, and oxidative stress.^40^ In NS, these mechanisms are particularly relevant, as hypoalbuminemia and dyslipidemia are well-known complications,^41^ and NS is associated with a proinflammatory state with elevated fibrinogen, IL6, and TNF*α*.^42^ Consistent with previous research, our findings support the association between NS and endothelial activation and injury. A study of 164 patients with NS reported elevated levels of vWF, syndecan-1, and e-selectin, with significantly higher vWF levels in those who developed VTE.^9^ Similarly, elevated thrombomodulin and E-selectin in patients with NS, further supporting the presence of endothelial activation and injury in this population.^8^ Endothelial integrity plays a crucial role in maintaining vascular homeostasis, and its disruption fosters a prothrombotic state by impairing anticoagulant mechanisms and promoting coagulation activation.^43^ In NS, endothelial dysfunction may further amplify hypercoagulability. Exposure to inflammation, oxidative stress, dyslipidemia, and hypoalbuminemia, all common in NS, can compromise endothelial function and downregulate protective mechanisms such as the antithrombin and protein C pathways.^40^ These changes may also impair fibrinolysis through reduced plasmin generation or increased expression of fibrinolytic inhibitors, consistent with our observation of prolonged clot lysis time.^44^

International guidelines recommend LMWH or warfarin to NS patients at high risk of VTE or, alternatively, aspirin in patents with increased bleeding risk.^15^ We observed no significant changes in platelet function in patients with NS when compared with healthy individuals, and while some studies have reported increased platelet aggregation in NS,^12,45^ our findings suggest that aspirin targeting platelet aggregation may not be effective as prophylaxis against VTE in patients with NS. This is supported by previous cohort studies showing that 46% of patients with NS who developed VTE were in fact receiving aspirin^6^ and that reduced effect of aspirin is a frequent finding in patients with NS.^21^

This study has several strengths. It provides a comprehensive characterization of the coagulation profile in NS patients, integrating assessments of endothelial cell function, platelet function, thrombin generation, and fibrinolysis in a single cohort. The use of clearly defined inclusion criteria ensures a well-characterized study population. Furthermore, the inclusion of patients with severe NS and a mean plasma albumin level of only 22 g/L suggest that this is a high-risk population for VTE. However, the study also has limitations. First, the cross-sectional design precludes evaluation of the contribution of these factors to adverse clinical outcomes such as VTE. Second, comparisons were made to different reference groups, and demographic data were not uniformly available across these cohorts, which limited the possibility of performing adjusted analyses between patients and controls. Third, the use of uACR >2200 mg/g as an inclusion criterion, while consistent with nephrotic-range proteinuria, may not fully capture the variability in proteinuria patterns across all NS subtypes. Fourth, exclusion of patients already on anticoagulant therapy may have introduced selection bias, as these individuals likely represent a very high-risk population. Fifth, although the sample size was sufficient to detect overall biomarker differences, it did not allow for subgroup comparisons across glomerular disease subtypes or evaluation of the potential impact of individual medications. Finally, platelet function was assessed using impedance aggregometry and TXB_2_, with both TXB_2_ and TAT offering relevant *in vivo* perspectives on platelet and thrombin-related activity. An additional marker such as P-selectin or *β*-thromboglobulin may have provided further insight. Finally, although endothelial cell markers were elevated, direct measures of vascular function were not included. Future studies should incorporate functional assessments, such as flow-medicated dilation or endothelin-1, to further elucidate the clinical relevance of endothelial dysfunction in NS.

In conclusion, multiple, significant coagulation alterations are observed in patients with NS, including increased *in vivo* and *ex vivo* thrombin generation markers, prolonged fibrinolysis time, and endothelial cell activation and injury. The disruption of endothelial integrity may contribute to sustained thrombin generation and fibrinolytic impairment, further amplifying the hypercoagulable state. The findings question the clinical utility of aspirin for prevention of VTE in NS and align with current guideline recommendations suggesting that anticoagulant therapy targeting secondary hemostasis may be more appropriate than antiplatelet agents in this population.

## Supplementary Material

## Data Availability

Partial restrictions to the data and/or materials apply. Data from this study are stored in the European Union Drug Regulating Authorities Clinical Trials Database database. Access to additional anonymized data can be granted upon reasonable request to the corresponding author, subject to applicable regulations and approvals.

## Supplemental Material

Supplemental Figure 1. Reference groups used for comparison in the study.

Supplemental Table 1. Thrombin generation, fibrinolysis, endothelial cell markers, and platelet function in patients with nephrotic syndrome and healthy individuals.

Supplemental Table 2. Multivariable linear regression analyses of potential predictors of coagulation biomarkers.

Supplemental Table 3. Multivariable linear regression analyses of potential predictors of endothelial biomarkers.

Supplemental Table 4. FDR-adjusted *P* values for biomarker comparison.

Statistical Analysis Plan
