# Supplementary material for: Alterations in Coagulation and Endothelial Function in Nephrotic Syndrome: A Multicenter, Cross-Sectional Analysis
Source: Kidney360. 2025 Jun 6;6(11):1960–9. doi: 10.34067/KID.0000000865 (PMC12626668; doi:10.34067/KID.0000000865)
Supplement: Supplementary file 1 [file kidney360-6-1960-s001.pdf]

Supplemental Materials

Alterations in Coagulation and Endothelial Function in Nephrotic Syndrome: A Multi-Center, Cross-Sectional Analysis

Sarah Kelddal[1,2], Erik L. Grove[3,4], Camilla L. Duus[5], Louis B. Nygaard[6], Tilde Kristensen[4,7], Frank H. Mose[4,6], Jon W. Gregersen[6], Anne-Mette Hvas[8], Henrik Birn[1,2,4]

[1]Department of Renal Medicine, Aarhus University Hospital, Aarhus, Denmark

[2]Department of Biomedicine, Aarhus University Health, Aarhus, Denmark

[3]Department of Cardiology, Aarhus University Hospital, Aarhus, Denmark

[4]Department of Clinical Medicine, Faculty of Health, Aarhus University, Aarhus, Denmark

[5]University Clinic in Nephrology and Hypertension, Goedstrup Hospital, Herning, Denmark

[6]Department of Nephrology, Aalborg University Hospital, Aalborg, Denmark

[7]Medical Diagnostics Centre, Viborg Regional Hospital, Viborg, Denmark

[8]Faculty of Health, Aarhus University, Aarhus, Denmark

Corresponding Author:

First and last name: Sarah Kelddal

E-mail address: kelddal@biomed.au.dk

Contents

Supplemental Figure 1 3

Figure 1 | Reference groups used for comparison in the study 3

Supplemental Table 1 4

Table 1 | Thrombin generation, fibrinolysis, endothelial cell markers, and platelet function in patients with nephrotic syndrome and healthy individuals 4

Supplemental Table 2 5

Multivariable linear regression analyses for predictors of coagulation and endothelial biomarkers 5

Supplemental Table 3 7

False Discovery Rate (FDR)-adjusted p-values for biomarker comparison 7

Statistical Analysis Plan 8

Supplemental Figure 1

Figure 1 | Reference groups used for comparison in the study

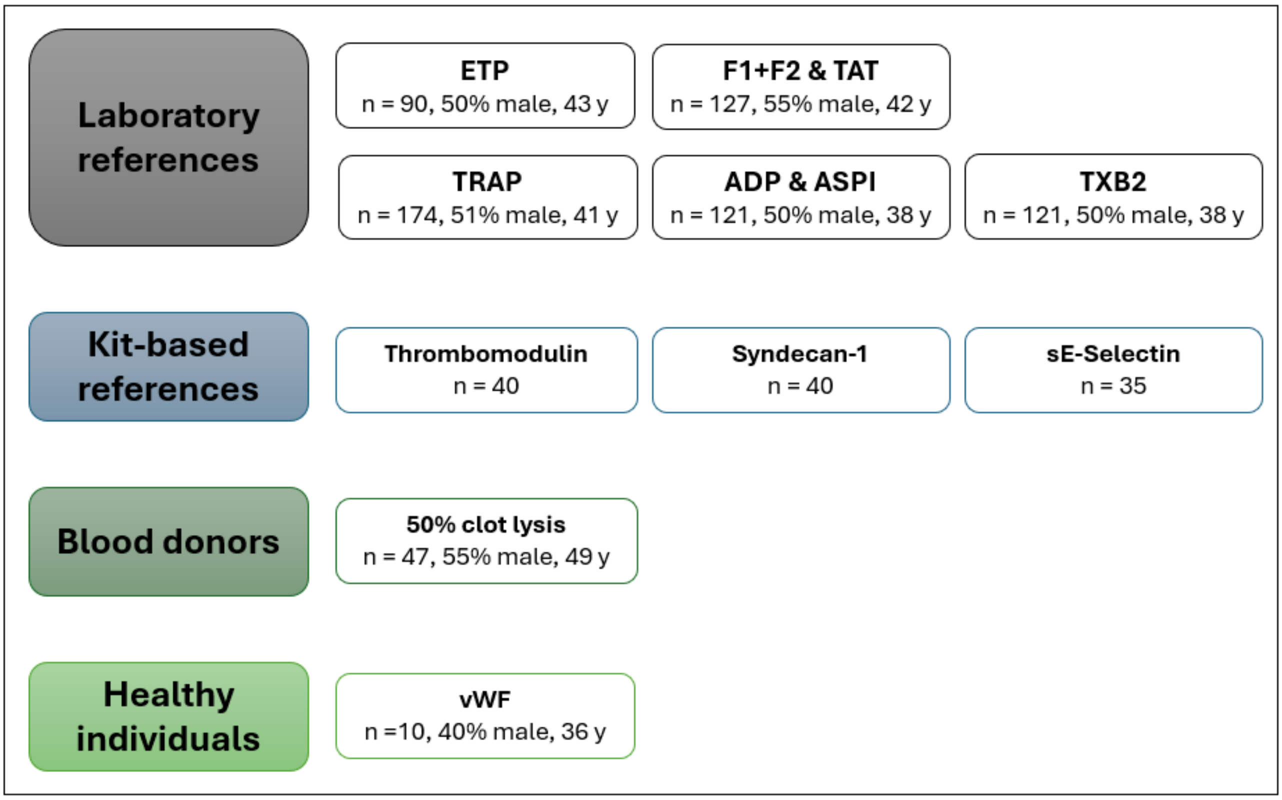

Reference groups used for comparison in this study. The groups are categorized based on the source of reference data: laboratory references, kit-based references, blood donor, and healthy individuals. The number of participants, gender distribution, and mean age are presented for each reference group. Demographic data were only partially available for the reference cohorts, and no full covariate adjustment could be performed in direct comparisons with patients.

Abbreviations: ETP: Endogenous thrombin potential, F1+F2: Prothrombin fragment 1+2, TAT: Thrombin-antithrombin complex, TRAP: Thrombin receptor activating peptide induced platelet aggregation, ADP: Adenosine diphosphate induced platelet aggregation, ASPI: Arachidonic acid induced platelet aggregation, TXB2: Thromboxane B_2, vWF: von Willebrand Factor.

Supplemental Table 1

Table 1 | Thrombin generation, fibrinolysis, endothelial cell markers, and platelet function in patients with nephrotic syndrome and healthy individuals

Variable	Patients with NS (n=47)	Healthy Individuals
**Thrombin generation markers, mean (95% CI)**		
Endogenous thrombin potential (nM x min)	1398 (1277-1519)	1281 (1237-1325)
Prothrombin fragment 1+2 (pmol/l)	509 (426-592)	183 (154-188)
Thrombin-antithrombin complex (µmol/l)	3.5 (3.2-3.8)	2.5 (2.3-2.8)
**Fibrinolysis marker, mean (95% CI)**		
50% clot lysis (s)	1281 (1101-1462)	1076 (903-1248)
**Endothelial cell markers, mean (95% CI)**		
Thrombomodulin (ng/ml)	30.2 (23.6-34.0)	5.1 (4.6-5.6)
Syndecan-1 (ng/ml)	91.9 (77.3-106.5)	31.6 (26.7-36.5)
sE-Selectin (ng/ml)	39.7 (33.6-45.8)	29.6 (26.4-32.8)
Von Willebrand factor 10^3 IU/l	3.0 (2.7-3.3)	1.38 (1.3-1.5)
**Platelet function, mean (95% CI)**		
TRAP (AU x min)	1231 (1125-1337)	1236 (1206-1266)
ADP (AU x min)	893 (779-1007)	810 (778-841)
ASPI (AU x min)	1013 (912-1113)	1004 (974-1034)

Values are presented as mean (95% confidence interval).
TRAP (Thrombin Receptor Activating Peptide), ADP (Adenosine Diphosphate), and ASPI (Arachidonic Acid) represent platelet aggregation responses measured in area under the curve (AU x min).

Supplemental Table 2

Multivariable linear regression analyses of potential predictors of coagulation biomarkers

**Outcome** (Dependent variable)	**Predictor** (Independent variable)	**β coefficient**	**95% CI**	**p-value**
Prothrombin fragment 1+2	Plasma albumin (g/L)	12.76	[–2.18, 27.71]	0.09
	uACR (mg/g)	–0.03	[–0.07, 0.01]	0.16
	eGFR (mL/min/1.73 m^2)	–4.88	[–7.91, –1.86]	0.002
	BMI (kg/m^2)	–4.92	[–19.22, 9.39]	0.94
	CRP (mg/L)	4.41	[–8.68,17.51]	0.50
	Age (years)	7.21	[3.32,11.09]	0.001
	Male (vs. female)	–114	[–279, 51]	0.17
Thrombin-antithrombin complex	Plasma albumin (g/L)	12.76	[–2.18, 27.71]	0.09
	uACR (mg/g)	–0.00008	[–0.0002, 0.00007]	0.27
	eGFR (mL/min/1.73 m^2)	–0.009	[–0.022, 0.004]	0.17
	BMI (kg/m^2)	–0.002	[–0.06, 0.06]	0.94
	CRP (mg/L)	–0.02	8–0.07, 0.03]	0.51
	Age (years)	0.01	[–0.007, 0.029]	0.23
	Male (vs. female)	–114	[–279, 51]	0.17
Endogenous thrombin potential	Plasma albumin (g/L)	–22.08	[–44.20, 0.04]	0.050
	uACR (mg/g)	0.02	[–0.04, 0.07]	0.51
	eGFR (mL/min/1.73 m^2)	4.08	[–0.77, 8.94]	0.10
	BMI (kg/m^2)	8.89	[–12.82, 30.61]	0.41
	CRP (mg/L)	3.76	[–16.18, 23.71]	0.71
	Age (years)	0.24	[–6.18, 6.66]	0.94
	Male (vs. female)	–222	[–458, 14]	0.06
50% clot lysis time	Plasma albumin (g/L)	28.74	[–2.85, 60.33]	0.07
	uACR (mg/g)	0.02	[–0.07, 0.10]	0.71
	eGFR (mL/min/1.73 m^2)	3.66	[–3.96, 11.28]	0.34
	BMI (kg/m^2)	–2.06	[–33.92, 29.81]	0.90
	CRP (mg/L)	–1.83	[–27.35, 23.68]	0.89
	Age (years)	3.66	[–6.34, 13.66]	0.46
	Male (vs. female)	37.7	[–337, 414]	0.84
ADP	Plasma albumin (g/L)	–20.42	[–41.91, 1.07]	0.06
	uACR (mg/g)	–0.004	[–0.06, 0.06]	0.90
	eGFR (mL/min/1.73 m^2)	1.86	[–4.14, 7.85]	0.53
	BMI (kg/m^2)	5.09	[–13.77, 23.94]	0.58
	CRP (mg/L)	5.45	[–8.94, 19.84]	0.44
	Age (years)	–0.16	[–5.66, 5.34]	0.95
	Male (vs. female)	–4.5	[–242, 233]	0.97

Each biomarker was analyzed as a dependent variable in a separate linear regression model, adjusting for plasma albumin (g/L), urine albumin-creatinine ration (uACR, mg/g), estimated glomerular filtration rate (eGFR, mL/min/1.73 m^2), body mass index (BMI, kg/m^2), and C-reactive protein (CRP, mg/L). The β coefficients indicate the expected change in the dependent variable per unit increase in the predictor variable. 95% confidence intervals (CI) and p-values are provided. A two-sided p-value < 0.05 was considered statistically significant.

Supplemental Table 3

Multivariable linear regression analyses of potential predictors of endothelial biomarkers

**Outcome** (Dependent variable)	**Predictor** (Independent variable)	**β coefficient**	**95% CI**	**p-value**
Thrombomodulin	Plasma albumin (g/L)	0.02	[–0.68, 0.72[]	0.95
	uACR (mg/g)	–0.0001	[–0.002, 0.002]	0.88
	eGFR (mL/min/1.73 m^2)	-0.20	[–0.34, 0.06]	0.006
	BMI (kg/m^2)	0.006	[–0.65, 0.67]	0.97
	CRP (mg/L)	–0.48	[–1.10, 0.14]	0.13
	Age (years)	5.48	[–1.84, 12.81]	0.14
	Male (vs. female)	–0.10	[–0.30, 0.09]	0.30
Syndecan-1	Plasma albumin (g/L)	–5.25	[–9.31, –1.19]	0.012
	uACR (mg/g)	0.002	[–0.01, 0.01]	0.75
	eGFR (mL/min/1.73 m^2)	0.55	[–0.37, 1.46]	0.24
	BMI (kg/m^2)	–2.41	[–6.42, 1.59]	0.23
	CRP (mg/L)	0.17	[–3.73, 4.08]	0.93
	Age (years)	0.04	[–0.29, 0.37]	0.82
	Male (vs. female)	7.02	[–5-28, 19.31]	0.26
sE-Selectin	Plasma albumin (g/L)	0.32	[–0.83, 1.49]	0.57
	uACR (mg/g)	0.001	[–0.001, 0.004]	0.32
	eGFR (mL/min/1.73 m^2)	–0.14	[–0.39, 0.11]	0.25
	BMI (kg/m^2)	0.69	[–0.39, 1.76]	0.21
	CRP (mg/L)	–0.14	[–1.49, 0.91]	0.79
	Age (years)	–1.64	[–2.76, –0.52]	0.005
	Male (vs. female)	21.90	[–24.46, 68.26]	0.35
von Willebrand factor	Plasma albumin (g/L)	–0.02	[–0.07, 0.04]	0.58
	uACR (mg/g)	-1.20×10^{-6}	[–0.0001, 0.0001]	0.99
	eGFR (mL/min/1.73 m^2)	0.004	[–0.01, 0.02]	0.50
	BMI (kg/m^2)	–0.03	[–0.05, 0.024]	0.27
	CRP (mg/L)	–0.008	[–0.06, 0.05]	0.75
	Age (years)	0.008	[–0.009, 0.024]	0.35
	Male (vs. female)	–0.19	[–0.82, 0.43]	0.54

Each biomarker was analyzed as a dependent variable in a separate linear regression model, adjusting for plasma albumin (g/L), urine albumin-creatinine ration (uACR, mg/g), estimated glomerular filtration rate (eGFR, mL/min/1.73 m^2), body mass index (BMI, kg/m^2), and C-reactive protein (CRP, mg/L). The β coefficients indicate the expected change in the dependent variable per unit increase in the predictor variable. 95% confidence intervals (CI) and p-values are provided. A two-sided p-value < 0.05 was considered statistically significant.

Supplemental Table 4

False Discovery Rate (FDR)-adjusted p-values for biomarker comparison

Biomarker	Group	Unadjusted p-value	FDR (group-wise)	FDR (global)
F1+F2	Thrombin	0.0010	0.0020	0.0028
TAT	Thrombin	0.0001	0.0002	0.0006
ETP	Thrombin	0.0127	0.0127	0.0175
50% clot lysis	Fibrinolysis	0.0040	0.0040	0.0073
TRAP	Platelet	0.9137	0.9137	0.9137
ADP	Platelet	0.0564	0.1692	0.0689
ASPI	Platelet	0.8334	1.0	0.9167
Thrombomodulin	Endothelial	0.0001	0.0004	0.0004
eS-selectin	Endothelial	0.0085	0.0085	0.0134
Syndecan-1	Endothelial	0.0001	0.0002	0.0011
vWF	Endothelial	0.0010	0.0013	0.0022

P-values calculated for group comparisons of key biomarkers related to thrombin generation, platelet function, endothelial injury, and fibrinolysis. To account for multiple comparisons, False Discovery Rate (FDR) correction was applied using the Benjamini-Hochberg procedure. Group-wise FDR correction was performed within biologically related categories (thrombin generation, platelet function, and endothelial markers) as well as a global FDR correction across all tests including 50% clot lysis time, which was not part of a multi-marker group. Both unadjusted and adjusted p-values are presented.

Statistical Analysis Plan

Manuscript Title: ***Alterations in Coagulation and Endothelial Function in Nephrotic Syndrome: A Multi-Center, Cross-Sectional Analysis***

Study Registration Number:

- Danish Medicines Agency (reference no.: 2020061178)
- Danish Research Ethics Committees (reference no.: 1-10-72-158-20)
- EudraCT (Identifier: 2019-001212-29)
- ClinicalTrials.gov (Identifier: NCT04850378)

Study Populations:

- **Nephrotic syndrome group:** Adults (≥ 18 years), plasma-albumin < 30 g/L, and uACR ≥ 2200 mg/g.
- **Healthy reference groups:** Used for selected markers; based on matched healthy donors, previously published laboratory dataset or manufacturer reference ranges.
- No individual-level matching was performed. However, for clot lysis assay, healthy reference samples were frequency-matched to nephrotic syndrome group based on age and gender.

Endpoints

Primary endpoints

- *In vivo* thrombin generation: F1+F2 and TAT.
- *Ex vivo* thrombin generation: ETP (AUC).
- Fibrinolytic activity: 50% clot lysis.

Secondary endpoints

- Endothelial markers: thrombomodulin, syndecan-1, sE-selectin, vWF.
- Platelet function: TRAP, ADP, ASPI, TXB2.
- Natural anticoagulants (antithrombin, protein C, free protein S).

Laboratory Methods

All blood samples were collected following standardized pre-analytical protocols. Citrated plasma was used for thrombin generation, fibrinolysis, and endothelial markers, with storage at –80°C prior to batch analysis. Whole blood in hirudin tubes was used for platelet aggregometry. All laboratory procedures followed validated assay protocols as detailed in the manuscript.

**Statistical Analyses**	**General approach**<br>- Data assessed for normality using QQ-plots.<br>- Continuous data: mean ± SD or median (IQR).<br>- Categorical data: counts and percentages.<br>- Missing values: no imputation; complete case analysis per variable.<br><br>**Group comparisons**<br>- Unpaired t-test for normally distributed variables (mean difference and 95% CI).<br>- Mann-Whitney U test for non-normally distributed variables (median and IQR).<br>- Paired t-test for within-subject comparisons (e.g., dalteparin vs. apixaban).<br>- Dot plots were used when individual values were available.<br><br>**Regression Analyses**<br>- Multivariable linear regression models were performed to adjust for potential confounders including p-albumin, uACR, eGFR, BMI, age, and gender.<br><br>**Multiple comparisons**<br>False discovery rate (FDR) correction was applied using the Benjamini-Hochberg procedure:<br>- Group-wise correction: within thrombin generation, platelet function, and endothelial markers.<br>- Global correction: across all biomarkers including 50% clot lysis time.
**Missing Data**	Analyses were conducted using available data only. Patients with missing data were excluded from the relevant analyses. No imputation was performed.
**Statistical Software**	- **Stata version 17.0** (StataCorp, TX, USA) used for all analyses.<br>- **GraphPad Prism version 10.3.1** (GraphPad Software, CA, USA) used for all figures.

Abbreviations	Explanations
	- ADP: Adenosine diphosphate<br>- ASPI: Arachidonic acid-induced platelet aggregation<br>- BMI: Body mass index<br>- CI: Confidence interval<br>- CRP: C-reactive protein<br>- eGFR: Estimated glomerular filtration rate<br>- ETP: Endogenous thrombin potential<br>- F1+F2: prothrombin fragment 1+2<br>- FDR: False discovery rate<br>- IQR: Interquartile range<br>- NS: Nephrotic syndrome<br>- PAI-1: Plasminogen activator inhibitor-1<br>- SD: Standard deviation<br>- TAT: Thrombin-antithrombin complex<br>- TRAP: Thrombin receptor-activating peptide<br>- TXB2: Thromboxane B2<br>- uACR: Urine albumin-to-creatinine ratio<br>- vWF: von Willebrand factor

Approval and Version History

Version	Date	Author	Notes
1.0	April 2025	Sarah Kelddal	Initial version submitted as supplement
