# Supplementary material for: Alterations in Coagulation and Endothelial Function in Nephrotic Syndrome: A Multicenter, Cross-Sectional Analysis
Source: Kidney360. 2025 Jun 6;6(11):1960–9. doi: 10.34067/KID.0000000865 (PMC12626668; doi:10.34067/KID.0000000865)
Supplement: Supplementary file 2 [file kidney360-6-1960-s002.pdf]

ASN American Society of Nephrology | JASN Journal of the American Society of Nephrology | KIDNEY360 Accessing Our World From Every Angle | CJASN Clinical Journal of the American Society of Nephrology

ASN Journal Disclosure Form

As per ASN journal policy, I have disclosed any financial relationships or commitments I have held in the past 36 months as included below. I have listed my Current Employer below to indicate there is a relationship requiring disclosure. If no relationship exists, my Current Employer is not listed.

H. Birn reports the following:
Employer: Department of Clinal Medicine, Aarhus University, Denmark, and Department of Nephrology, Aarhus University Hospital, Denmark.; Consultancy: NOVO Nordisk, Astra Zeneca; Galapagos, Boehringer Ingelheim, Bayer, Glaxo Smith Kline (GSK);; Research Funding: Glaxo Smith Kline (GSK); Vifor Pharma; NOVO Nordisk; Honoraria: Astra Zeneca; Boehringer Ingelheim, Bayer, MSD; NOVO Nordisk;; Patents or Royalties: Aarhus University Business and Innovation; Advisory or Leadership Role: Danish Society of Nephrology; Kidney and Dialysis; Danish Health Authority; Augustinus Foundation; Helen and Ejnar Bjørnow Foundation;; and Speakers Bureau: BestPractice Nordic (medical information service); Dagens Medicin A/S; Danish Kidney Association.

I understand that the information above will be published within the journal article, if accepted, and that failure to comply and/or to accurately and completely report the potential financial conflicts of interest could lead to the following: 1) Prior to publication, article rejection, or 2) Post-publication, sanctions ranging from, but not limited to, issuing a correction, reporting the inaccurate information to the authors' institution, banning authors from submitting work to ASN journals for varying lengths of time, and/or retraction of the published work.

Name: Henrik Birn
Manuscript ID: K360-2025-000344R1
Manuscript Title: Alterations in Coagulation and Endothelial Function in Nephrotic Syndrome: A Multi-Centre, Cross-Sectional Analysis
Date of Completion: May 14, 2025
Disclosure Updated Date: May 14, 2025

ASN American Society of Nephrology | JASN Journal of the American Society of Nephrology | KIDNEY360 Accessing Our World From Every Angle | CJASN Clinical Journal of the American Society of Nephrology

ASN Journal Disclosure Form

As per ASN journal policy, I have disclosed any financial relationships or commitments I have held in the past 36 months as included below. I have listed my Current Employer below to indicate there is a relationship requiring disclosure. If no relationship exists, my Current Employer is not listed.

C. Duus reports the following:
Employer: University Clinic in Nephrology and Hypertension, Regional Hospital Gødstrup and Aarhus University, Denmark

I understand that the information above will be published within the journal article, if accepted, and that failure to comply and/or to accurately and completely report the potential financial conflicts of interest could lead to the following: 1) Prior to publication, article rejection, or 2) Post-publication, sanctions ranging from, but not limited to, issuing a correction, reporting the inaccurate information to the authors' institution, banning authors from submitting work to ASN journals for varying lengths of time, and/or retraction of the published work.

Name: Camilla Lundgreen Duus
Manuscript ID: K360-2025-000344R1
Manuscript Title: Alterations in Coagulation and Endothelial Function in Nephrotic Syndrome: A Multi-Centre, Cross-Sectional Analysis
Date of Completion: April 29, 2025
Disclosure Updated Date: April 29, 2025

ASN Journal Disclosure Form

As per ASN journal policy, I have disclosed any financial relationships or commitments I have held in the past 36 months as included below. I have listed my Current Employer below to indicate there is a relationship requiring disclosure. If no relationship exists, my Current Employer is not listed.

J. Gregersen reports the following:
Employer: Aalborg University Hospital; Consultancy: Vifor Pharma;; and Advisory or Leadership Role: Vifor Pharma, Otsuka.

I understand that the information above will be published within the journal article, if accepted, and that failure to comply and/or to accurately and completely report the potential financial conflicts of interest could lead to the following: 1) Prior to publication, article rejection, or 2) Post-publication, sanctions ranging from, but not limited to, issuing a correction, reporting the inaccurate information to the authors' institution, banning authors from submitting work to ASN journals for varying lengths of time, and/or retraction of the published work.

Name: Jon Waarst Gregersen
Manuscript ID: K360-2025-000344R1
Manuscript Title: Alterations in Coagulation and Endothelial Function in Nephrotic Syndrome: A Multi-Centre, Cross-Sectional Analysis,
Date of Completion: May 18, 2025
Disclosure Updated Date: May 18, 2025

ASN American Society of Nephrology | JASN Journal of the American Society of Nephrology | KIDNEY360 Accessing Our World From Every Angle | CJASN Clinical Journal of the American Society of Nephrology

ASN Journal Disclosure Form

As per ASN journal policy, I have disclosed any financial relationships or commitments I have held in the past 36 months as included below. I have listed my Current Employer below to indicate there is a relationship requiring disclosure. If no relationship exists, my Current Employer is not listed.

E. Grove reports the following:
Employer: Aarhus University Hospital; Consultancy: ELG has previously received consultancy fees from Bayer and Bristol Myers-Squibb. ; No ongoing or planned activities.; Ownership Interest: Novo Nordisk; Research Funding: Unrestricted research grant from Boehringer Ingelheim.; Investigator in studies sponsored by AstraZeneca, Idorsia and Bayer.; Honoraria: ELG has previously received Lecture fees received from Bayer, Pfizer, and Bristol-Myers Squibb.; No ongoing or planned activities.; Advisory or Leadership Role: Novo Nordisk; and Other Interests or Relationships: Unpaid activities: Chair of the Danish Society of Thrombosis & Haemostasis + Nucleus member of WG on Cardiovascular Pharmacotherapy (European Society of Thrombosis and Haemostasis).

I understand that the information above will be published within the journal article, if accepted, and that failure to comply and/or to accurately and completely report the potential financial conflicts of interest could lead to the following: 1) Prior to publication, article rejection, or 2) Post-publication, sanctions ranging from, but not limited to, issuing a correction, reporting the inaccurate information to the authors' institution, banning authors from submitting work to ASN journals for varying lengths of time, and/or retraction of the published work.

Name: Erik Lerkevang Grove
Manuscript ID: K360-2025-000344R1
Manuscript Title: Alterations in Coagulation and Endothelial Function in Nephrotic Syndrome: A Multi-Centre, Cross-Sectional Analysis
Date of Completion: April 30, 2025
Disclosure Updated Date: April 30, 2025

ASN American Society of Nephrology
JASN Journal of the American Society of Nephrology
KIDNEY360 Accessing Our World From Every Angle
CJASN Clinical Journal of the American Society of Nephrology

ASN Journal Disclosure Form

As per ASN journal policy, I have disclosed any financial relationships or commitments I have held in the past 36 months as included below. I have listed my Current Employer below to indicate there is a relationship requiring disclosure. If no relationship exists, my Current Employer is not listed.

A. Hvas reports the following:
Employer: Aarhus University

I understand that the information above will be published within the journal article, if accepted, and that failure to comply and/or to accurately and completely report the potential financial conflicts of interest could lead to the following: 1) Prior to publication, article rejection, or 2) Post-publication, sanctions ranging from, but not limited to, issuing a correction, reporting the inaccurate information to the authors' institution, banning authors from submitting work to ASN journals for varying lengths of time, and/or retraction of the published work.

Name: Anne-Mette Hvas
Manuscript ID: K360-2025-000344R1
Manuscript Title: Alterations in Coagulation and Endothelial Function in Nephrotic Syndrome
Date of Completion: May 15, 2025
Disclosure Updated Date: May 15, 2025

ASN American Society of Nephrology
JASN Journal of the American Society of Nephrology
KIDNEY360 Accessing Our World From Every Angle
CJASN Clinical Journal of the American Society of Nephrology

ASN Journal Disclosure Form

As per ASN journal policy, I have disclosed any financial relationships or commitments I have held in the past 36 months as included below. I have listed my Current Employer below to indicate there is a relationship requiring disclosure. If no relationship exists, my Current Employer is not listed.

S. Kelddal reports the following:
Employer: Department of Biomedicine, Aarhus University, Denmark and Department of Nephrology, Aarhus University Hospital; and Research Funding: I have obtained funding from the Danish Kidney Association 2023 and the Augustinus Foundation.

I understand that the information above will be published within the journal article, if accepted, and that failure to comply and/or to accurately and completely report the potential financial conflicts of interest could lead to the following: 1) Prior to publication, article rejection, or 2) Post-publication, sanctions ranging from, but not limited to, issuing a correction, reporting the inaccurate information to the authors' institution, banning authors from submitting work to ASN journals for varying lengths of time, and/or retraction of the published work.

Name: Sarah Kelddal
Manuscript ID: K360-2025-000344R1
Manuscript Title: Alterations in Coagulation and Endothelial Function in Nephrotic Syndrome: A Multi-Centre, Cross-Sectional Analysis
Date of Completion: April 30, 2025
Disclosure Updated Date: April 30, 2025

ASN American Society of Nephrology | JASN Journal of the American Society of Nephrology | KIDNEY360 Accessing Our World From Every Angle | CJASN Clinical Journal of the American Society of Nephrology

ASN Journal Disclosure Form

As per ASN journal policy, I have disclosed any financial relationships or commitments I have held in the past 36 months as included below. I have listed my Current Employer below to indicate there is a relationship requiring disclosure. If no relationship exists, my Current Employer is not listed.

T. Kristensen reports the following:
Employer: Regional Hospital Viborg; Research Funding: Augustinus Foundation; and Honoraria: Teeching Fee from AstraZeneca.

I understand that the information above will be published within the journal article, if accepted, and that failure to comply and/or to accurately and completely report the potential financial conflicts of interest could lead to the following: 1) Prior to publication, article rejection, or 2) Post-publication, sanctions ranging from, but not limited to, issuing a correction, reporting the inaccurate information to the authors' institution, banning authors from submitting work to ASN journals for varying lengths of time, and/or retraction of the published work.

Name: Tilde Kristensen
Manuscript ID: K360-2025-000344R1
Manuscript Title: "Alterations in Coagulation and Endothelial Function in Nephrotic Syndrome: A Multi-Centre, Cross-Sectional Analysis,"
Date of Completion: April 29, 2025
Disclosure Updated Date: April 29, 2025

ASN American Society of Nephrology | JASN Journal of the American Society of Nephrology | KIDNEY360 Accessing Our World From Every Angle | CJASN Clinical Journal of the American Society of Nephrology

ASN Journal Disclosure Form

As per ASN journal policy, I have disclosed any financial relationships or commitments I have held in the past 36 months as included below. I have listed my Current Employer below to indicate there is a relationship requiring disclosure. If no relationship exists, my Current Employer is not listed.

F. Mose reports the following:
Employer: Department of Medicine; Research Funding: Boehringer Ingelheim; Novo Nordisk; and Honoraria: Boehringer Ingelheim; Novo Nordisk.

I understand that the information above will be published within the journal article, if accepted, and that failure to comply and/or to accurately and completely report the potential financial conflicts of interest could lead to the following: 1) Prior to publication, article rejection, or 2) Post-publication, sanctions ranging from, but not limited to, issuing a correction, reporting the inaccurate information to the authors' institution, banning authors from submitting work to ASN journals for varying lengths of time, and/or retraction of the published work.

Name: Frank H. Mose
Manuscript ID: K360-2025-000344R1
Manuscript Title: Alterations in Coagulation and Endothelial Function in Nephrotic Syndrome: A Multi-Centre, Cross-Sectional Analysis
Date of Completion: May 30, 2025
Disclosure Updated Date: May 30, 2025

ASN American Society of Nephrology
JASN Journal of the American Society of Nephrology
KIDNEY360 Accessing Our World From Every Angle
CJASN Clinical Journal of the American Society of Nephrology

ASN Journal Disclosure Form

As per ASN journal policy, I have disclosed any financial relationships or commitments I have held in the past 36 months as included below. I have listed my Current Employer below to indicate there is a relationship requiring disclosure. If no relationship exists, my Current Employer is not listed.

L. Nygaard reports the following:
Honoraria: Speaking honoraria from CSL Vifor

I understand that the information above will be published within the journal article, if accepted, and that failure to comply and/or to accurately and completely report the potential financial conflicts of interest could lead to the following: 1) Prior to publication, article rejection, or 2) Post-publication, sanctions ranging from, but not limited to, issuing a correction, reporting the inaccurate information to the authors' institution, banning authors from submitting work to ASN journals for varying lengths of time, and/or retraction of the published work.

Name: Louis Nygaard
Manuscript ID: K360-2025-000344R1
Manuscript Title: Alterations in Coagulation and Endothelial Function in Nephrotic Syndrome: A Multi-Centre, Cross-Sectional Analysis
Date of Completion: May 31, 2025
Disclosure Updated Date: May 31, 2025
